# The Epidemiology of Hepatitis D Virus in North Africa: A Systematic Review and Meta-Analysis

**DOI:** 10.1155/2018/9312650

**Published:** 2018-09-26

**Authors:** Mohamed A. Daw, Amina M. Daw, Nadia E. M. Sifennasr, Aisha M. Draha, Ahmed M. Daw, Ali M. Daw, Mohamed O. Ahmed, Ebtisam S. Mokhtar, Abdallah El-Bouzedi, Ibrahem M. Daw

**Affiliations:** ^1^Department of Medical Microbiology & Immunology, Faculty of Medicine, University of Tripoli, CC 82668, Tripoli, Libya; ^2^Department of General Medicine, Faculty of Medicine, University of Tripoli, CC 82668, Tripoli, Libya; ^3^Department of Pharmacology, Faculty of Medicine, University of Tripoli, CC 82668, Tripoli, Libya; ^4^Tripoli Medical Centre, Faculty of Medicine, University of Tripoli, CC 82668, Tripoli, Libya; ^5^Department of Microbiology and Parasitology, Faculty of Veterinary Medicine, University of Tripoli, CC 82668, Tripoli, Libya; ^6^Department of Laboratory Medicine, Faculty of Biotechnology, University of Tripoli, CC 82668, Tripoli, Libya; ^7^Department of Planning, Faculty of Engineering, University of Tripoli, CC 82668, Tripoli, Libya

## Abstract

**Background:**

Hepatitis D virus (HDV) infection has been considered a serious neglected pandemic, particularly in developing countries. The virus causes a more severe disease than mono infection with hepatitis B virus (HBV). The epidemiology of HDV is not well documented in North Africa, which is known to be endemic for HBV. In this study, we explored the prevalence of HDV infection and also attempted to identify factors associated with hepatitis D positive status among chronic hepatitis B patients in North Africa.

**Methods:**

The electronic databases PubMed, Embase, Scopus, Science Direct, Web of Science, and Google Scholar were comprehensively searched for all papers published between January 1, 1998, and December 31, 2017, using appropriate strategies containing all related keywords, including North Africa, names of countries in the region, and all permutations of hepatitis D virus. The estimated prevalence of HDV in North Africa was calculated as an average of the pooled infection prevalence in each country weighted by the ratio of the country's hepatitis D virus population to the study's sample size in the survey data analysis.

**Findings:**

A total of 312 studies were identified and 32 were included in this study, with a total sample of 4907 individuals screened for HDV. There was considerable variability in the prevalence estimates of HDV within the countries of the region. The overall prevalence of HDV in the general population of North Africa was 5·01% (95% CI: 1·25–8·27) and in liver disease patients it was 20.7% (95% CI:9.87–44.53). Genotype-1 was the most prominent genotype reported in five published studies. Ten studies reported on HDV RNA in participants who were seropositive for HDV, and four studies highlighted the impact of demographic factors (sex and age). No study showed the impact of risk factors on the prevalence of HDV in North Africa.

**Interpretation:**

This review provides a comprehensive assessment of the burden of HDV in Northern Africa. There were significant differences in seroprevalence, study population, and diagnostic testing between the countries in the region. The results presented here will alert health professionals to implement clear policies based on evidence to diminish the burden of HDV infection. Such measures may include but are not restricted to improving the laboratory diagnostic tests and initiating patient data registries and blood screening. Further epidemiological and research studies are needed to explore the risk factors, coinfections, and approaches to increase testing for HDV, particularly in high-risk subpopulations, such as intravenous drug users and immigrants, and to define the consequences of HDV infection in North Africa.

## 1. Introduction

Hepatitis delta virus (HDV) is a unique RNA agent. It is the smallest infectious agent, and it causes the most severe form of viral hepatitis in humans. The viral genome consists of one single-stranded circular RNA molecule of about 1.7 kb with negative polarity. It is a new virus species originating from vertebrates and the only member of the Deltaviridae family (genus Deltavirus). The genome is similar to that of higher plant virusoids and virusoid RNA, which suggest that HDV might have originated from the plant instead of the animal world [1, 2]. Due to a large amount of base pairings, the viral RNA adopts a rod-like structure. The RNA encodes a protein called the delta antigen, which is subsequently packaged with hepatitis B surface antigen (HBsAg) and encased in an envelope [3, 4]. The HDV RNA is unique in its ability to self-cleave due to the presence of a ribozyme in the genomic and antigenomic sequences of its RNA. This sequence of about 85 nucleotides is capable of self-cleavage and self-binding [5, 6].

Different studies have shown that HDV infection aggravates the course of a hepatitis B virus (HBV) coinfection, accelerating progression to cirrhosis and leading to early decompensation of liver function compared with monoinfection with HBV [7, 8]. Epidemiological studies have shown that HDV is present worldwide and that its local prevalence is a function of the prevalence of HBV [9]. However, despite a large overlap, the prevalence of HDV does not always coincide with that of HBV [10].

Parenteral exposure to blood of infected individuals (needle-stick injuries, injection drug use, and transfusions) is the most efficient way of HDV transmission, particularly in HBsAg-positive individuals. Recent studies provide increasing evidence that sexual transmission (*via* semen or vaginal secretions) may be important in the spread of HDV infection. However, vertical transmission from mother to offspring, homosexual promiscuity, and nosocomial exposure seem to be inconspicuous risk factors [11, 12]. Most HDV-infected patients are either intravenous drug users or immigrants. HDV can be transmitted only in the existence of concomitant HBV infection in one of two patterns: simultaneous coinfection or HDV infection of an individual chronically infected with HBV (superinfection) [13, 14]. In developing countries, including those in North Africa, HDV infection is considered a healthcare-associated infection aggravated by needle-stick injury, use of contaminated syringes, and male health practice, in the absence of universal precautions for infection control. In the community, it is associated with pregnancy and cultural practices such as tattoos and circumcision.

It is estimated that over 20 million people are infected with HDV. The prevalence varies greatly from one region to another. The highest prevalence rates have been reported in the Mediterranean basin, the Middle East, central and northern Asia, sub-Saharan Africa, and South America [15, 16]. At least eight HDV genotypes have been described. Genotype I is the most prevalent and has been found in Europe, the Middle East, North America, and North Africa. Genotype II has been found in the Far East, Genotype III has been found in South America, and Genotypes IV–VIII have been found in Africa [17, 18]. HDV genotyping was found to be helpful in identifying patients with an increased or reduced risk of developing end-stage liver disease and may be considered for immigrants or populations with a high prevalence of a variety of genotypes [19, 20].

North African countries have a unique geographical location facing the European Union. They act as a focus of African immigrants heading to Europe [21]. HDV is considered endemic in this region and viral screening for HDV or specific clinical targeting is rarely practiced. However, there are no reliable data on its seroprevalence in North Africa. Therefore, the purpose of this review was to provide detailed data on the prevalence of HDV in North African populations and particularly in specific population groups such as blood donors and pregnant women. Furthermore, we aimed to identify the approaches needed to influence decision-making towards interventions aimed at reducing the burden of HDV in North African countries.

## 2. Methods

The data in this review are reported according to the guidelines of Preferred Reporting Items for Systematic Reviews and Meta-Analyses (PRISMA) [22, 23].

### 2.1. Geographic Setting

North Africa refers to the northernmost region of Africa and stretches from the Atlantic shores of the Western Sahara of Mauritania in the west to Egypt in the east. It comprises 35% of the area of Africa and consists of the three of the four largest countries in Africa: Algeria, Libya, and Sudan, together with Egypt, Tunisia, Morocco, and Mauritania. These countries are members of the Arab League, and their populations speak Arabic and share the same heritage, but they vary considerably in infrastructure, economic development, and social demography (Supplement 1). Viral hepatitis is considered endemic in this region. Importantly, Egypt has the highest prevalence of HCV in the world and Sudan and Mauritania have the highest prevalence of HBV.

### 2.2. Search Strategy and Selection Criteria

The search strategy consisted of a literature search in PubMed, Embase, Scopus, Science Direct, Web of Science, and Google Scholar for studies published between January 1, 1998, and December 31, 2017, in which the prevalence of anti-HDV antibody or HDV RNA was reported. Titles and/or abstracts were screened to determine the relevance of the studies. Full texts of the selected studies were reviewed. Data search strings included Northern Africa and the names of the countries therein: Algeria, Egypt, Libya, Mauritania, Morocco, Sudan, and Tunisia. We also collected data on detection of anti-HDV antibodies in HBsAg-positive individuals, geographical location, patient category, clinical setting, and HDV genotyping.

The references cited by all the selected original research articles and reviews were searched for additional articles that might have been missed. Any document reporting a measure of HDV epidemiology based on primary data was considered. Studies were excluded if they did not contain primary data, were on younger patients of nonspecified age, or used nonreferenced sources. We also excluded letters, commentaries, correspondence, editorials, and duplicate data (up-to-date version was used). Studies conducted in countries outside North Africa on immigrants from the North African countries were also excluded.

### 2.3. Data Extraction and Quality Assessment

All articles included in the analysis were reviewed by title and abstract, and then full-text was examined. The following data were recorded from each article using a standardized protocol: surname of the first author, study title, publication year, country where the study was conducted, prospective or retrospective nature of the study, method of HDV diagnosis, and number of patients with HDV infection. Specific attention was given to study design, study population characteristics, and serological evidence of HDV.

Critical Appraisal Checklist was applied to each paper to evaluate the adequacy of the sample size, study design, data collection, and the resultant presentation. Each paper was independently assessed by two authors for inclusion, and disagreements were resolved by consensus. Meta-analysis of HDV prevalence was conducted for each country and for the various populations at risk whenever data on the prevalence of HDV were available. The variation in the prevalence was stabilized using the Freeman-Tukey type arcsine square root transformation [24, 25].

### 2.4. Statistical and Biodata Analysis

Statistical analysis was carried out by calculating confidence intervals (CI), random-effects model, sensitivity analysis, and cut-off p-value (*p*= 0.1). The prevalence of HDV was calculated as the average of the pooled infection prevalence rates of each country weighted by the ratio of the country's HBV population to the study's sample size in the survey data analysis. Risk of bias in reporting the prevalence and cumulative incidence were independently calculated by the authors. Publication bias was assessed by inspecting a funnel plot and using Egger's test [17]. Analyses of aggregated prevalence of each country were done with metan command, in which the value is an average of the individual study results weighted by the inverse of their variances using a fixed/random model [26].

### 2.5. Ethical Approval

This study did not require ethical approval as it was based on data retrieved from studies already available in the public domain.

## 3. Results

**Figure 1:**
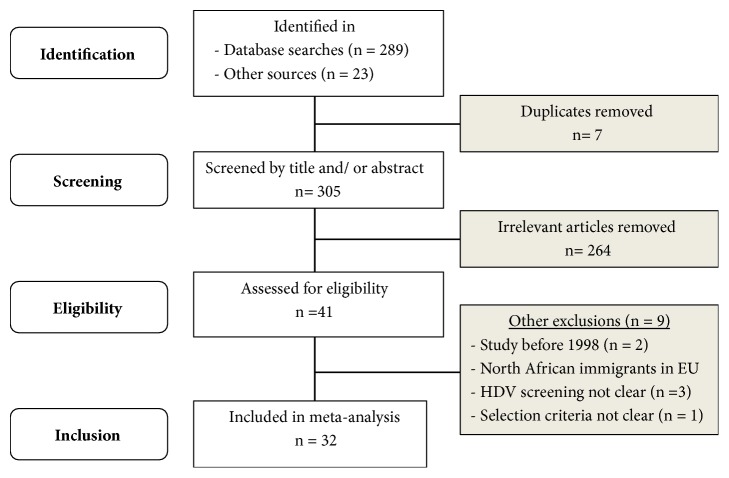
Flow chart of article selection for the systematic review and meta-analysis of hepatitis D virus epidemiology in North Africa (1998-2017).

A total of 312 studies were initially identified, of which 7 duplicates were eliminated and 264 were excluded because they were irrelevant and did not fulfill the inclusion criteria. Of the remaining 41 studies, nine others were discarded. Thus, after quality evaluation, only 32 studies, all of moderate qualities were included in the analysis (Figure 1). No paper or review article was found evaluating the prevalence of HDV in North Africa* perse* or highlighting the overall prevalence of HDV in any of the countries included in this review.

The 32 studies described 19 populations from the seven countries in North Africa: Egypt, Tunisia, Mauritania, Sudan, Libya, Algeria, and Morocco [16, 27–63]. The study populations were recruited from different settings: nine were from liver disease patients, seven were from hospital patients, five were from blood donors or the general populations, four from pregnant women, and seven were from other different groups (Table 1).

**Figure 2:**
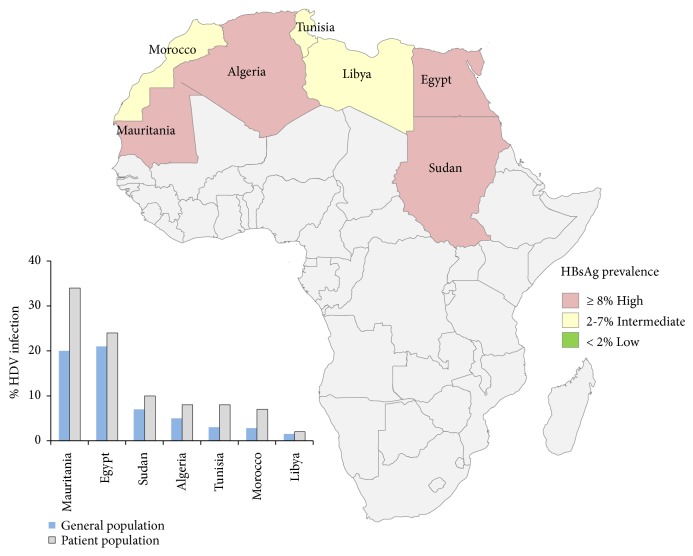
Seroepidemiology of hepatitis D virus in North African populations in studies published during 1998–2017.

**Figure 3:**
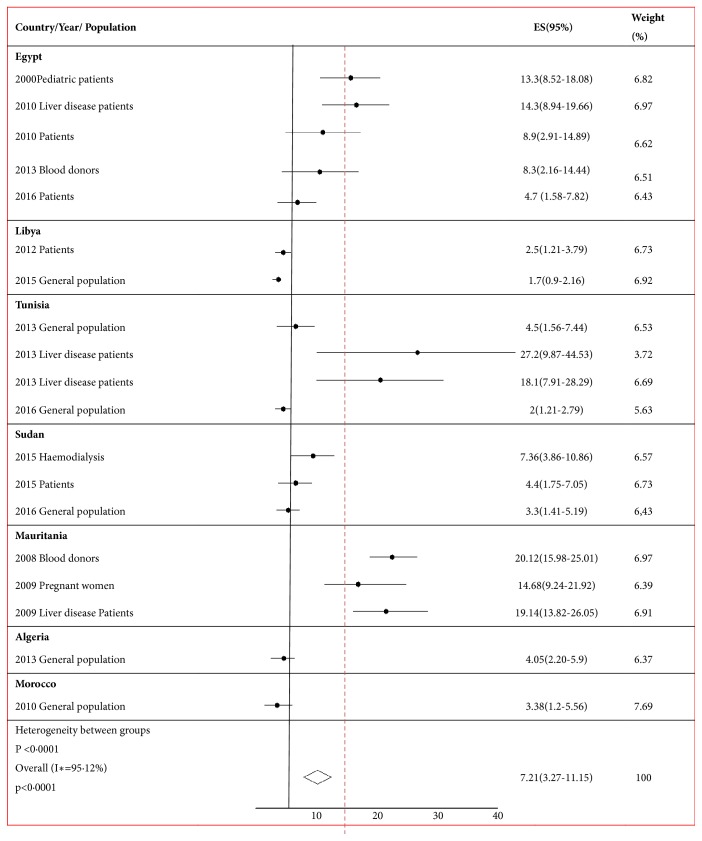
Forest plot of the seroprevalence of hepatitis D virus in different populations in North Africa.

The overall prevalence rates of HDV infection (HBsAg positive) in the general populations examined in the Northern Africa region ranged from 1.2 to 8.9%. The lowest rate was reported in Libya, followed by Tunisia, Algeria, and Morocco. The highest rates were reported in Mauritania, Sudan, and Egypt (Figure 2). The prevalence rate of HDV among liver disease and hemodialysis patients ranged from 19% to 27.2% with an estimated seroprevalence of 20.7% (95% CI: 9.87-44.53). Significant variability in HDV seroprevalence was noticed among the countries in the region, particularly among liver disease patients. In studies specially those reported from Egypt, Tunisia, Sudan, and Mauritania, in which patients with confirmed liver disease were compared with the general population (asymptomatic and without evidence of hepatic diseases), the presence of antihepatitis D virus among patients with liver diseases and HBsAg positive was statistically significant (*P*< 0.001) as illustrated in Figure 3.

Enzyme immunoassays were used to test for total anti-HDV and qualitative PCR was used to detect HDV RNA in patients who were seropositive for HDV. Of the total 162 individuals who were tested for both anti-HDV and HDV RNA; 98 (60%) were positive for HDV RNA. The largest populations tested were in Mauritania. These accounted for 121 cases, giving aHDV-RNA prevalence ranging from 69 to 79%, followed by Egypt (n = 18; 25-30%), Libya (n = 4; 25%), Sudan (n = 3; 30%), and Tunisia (n = 16; 30-33%). No studies on HDV RNA were done in Morocco or Algeria. RNA positivity among anti-DHV-positive individuals was reported to be high particularly in studies carried in Egypt, Sudan, and Mauritania.

The majority of the studies included adult populations but did not classify the prevalence by age groups. Only four studies showed that the prevalence varied statistically with age but without a clear trend for any specific age range. The highest prevalence was noticed in the age range of 18-45 years (14-45%). Only one study was carried out on children <16 years of age, but no statistically significant age-related difference was observed. Six studies examined the prevalence of HDV in males and females. A detailed analysis of these studies showed no relation of sex to HDV prevalence. Nevertheless, two studies showed a higher HDV prevalence among women, but the difference from men was not statistically significant. Only one study analyzed the HDV prevalence among married (8.1%) and single persons (3.7%).

Four studies on blood donors showed anti-HDV prevalence rates of 0-2.7%. Few studies were performed on children, showing an overall prevalence of 1.3%. Two studies examined HDV prevalence among pregnant women (n = 199). The HDV prevalence rates in these studies were 3.3% (95% CI: 1.41-5.19) and 15% (95% CI = 95% 9-23). The estimated combined prevalence was 6.2% (95% CI: 3.0–5.9).

No study comparing the prevalence of HDV between urban and rural communities was found. Only three studies included genotyping analysis: in Egypt, Tunisia, and Mauritania. HDV genotype I was the most prevalent in Egypt. Similar results were reported in Mauritania, as HDV genotype 1 accounted for over 90%, in addition to few strains of genotype 5. A study carried out on 32 patients infected with HDV in the northeastern coast of Tunisia showed that all HDV strains were genotype 1, with a wide distribution within the HDV-1 group. They all share the African amino acid marker, a serine at position 202 of the large delta protein.

## 4. Discussion

HDV infection is considered as a silent threat, particularly in developing countries. The virus can cause a severe form of chronic viral hepatitis that progresses to cirrhosis and increases the risk of liver cancer compared to hepatitis B and C viruses [64]. Epidemiological studies on the prevalence of HDV infection in North Africa are scant, and accurate data are needed to implement proper policies for prevention, diagnosis, and management of HDV infection [65].

This systematic review included 32 studies on the prevalence of HDV conducted over the past 20 years in North Africa. These studies include the data of 4,709 individuals. This review provides descriptive information on hepatitis D in the general populations and in blood donors, as well as in special subgroups, such as pregnant women and people with liver disease. The overall prevalence of anti-HDV among the general HBsAg-positive populations in North Africa was estimated at 5.1% (95% CI: 1.2–11.4), corresponding to about four million people infected with HDV in Egypt alone, and it may reach up to eleven millions in the whole region.

HDV prevalence varies greatly within the populations of each North African country. The highest rates were reported in Egypt, Sudan, and Mauritania, followed by Tunisia, Morocco, Algeria, and Libya. This should be interpreted with caution due to the small number of individuals tested and the quality of the testing methods. More data are needed, particularly from Morocco and Algeria, to confirm these findings. Similar results were reported in other geographical regions in Africa, including sub-Saharan and Central Africa, where such heterogeneity is evident. This may suggest clusters of endemicity in Africa [66, 67].

Demographic factors such as sex and age, which may influence the prevalence of HDV in North Africa, were analyzed in this review. HDV prevalence was reported consistent at all ages among adults of 18-45 years, but no specific age trend and no significant association with sex were observed. This is in agreement with a recent prospective multicenter study conducted in Taiwan indicating that there is a significantly increasing trend in HDV prevalence with age in people who are not intravenous drug users [68]. Blood donors and pregnant women have shown prevalence rates of infection similar to those of the general population. Furthermore, no study compared the prevalence of HDV in urban and rural regions in North Africa despite the great diversity in the level of urbanization in these countries. A recent study on HCV in this region showed that the prevalence of HCV in rural areas is higher than in urban cities, particularly in Egypt and Sudan [69].

HDV RNA was measured in participants positive for anti-HDV in only nine cohort studies, in which a total of 416 participants were tested. The prevalence of HDVRNA was consistent in most of the studies reported. In Egypt, Tunisia, Sudan, and Libya, HDVRNA prevalence was similar in the different subpopulations studied, including out-patients, blood donors, and pregnant women; it ranged from 25 to 30% but reached 69% in Mauritania. However, no data have been reported from Algeria and Morocco.

The prevalence of HDV in liver disease patients and in hemodialysis patients in North Africa was three to four times higher than in the general population and in blood donors, particularly in Egypt, Sudan, Mauritania, and Tunisia. This is in agreement with other studies in the Far East, South America, and sub-Saharan Africa [9, 70]. Such studies highlight the contribution of HDV to the burden of liver diseases such as fibrosis and cirrhosis. However, the data presented are insufficient to determine the influence of HDV on the consequences of liver diseases. Furthermore, data on hepatic liver diseases are insufficient in North Africa and are hampered by inadequate disease surveillance and lack of high-quality tools to assess chronic liver diseases. Hence, studies are needed to characterize and validate clinical measures of liver fibrosis in this region [71].

HDV strains are classified into eight distinct clades (1 to 8) that vary from one geographic region to another [72]. Studies on HDV genotyping in North Africa are scanty and only a few studies have been published, mainly from Egypt and Tunisia. HDV genotype I is the most dominant genotype in this region, accounting for 90% of the strains, with few strains of genotype 5. This is in agreement with other published reports from sub-Saharan and Central Africa. These genotypes appear to be geographically localized and to be variably associated with clinical presentation. Genotype 1 has been reported to have rapid and aggressive HDV virion formation and dissemination. Therefore, patients infected with this type have more adverse outcomes and decreased survival.

The number of HDV genotypes was not well documented in all countries and available data may not have captured the diversity and true frequency of HDV genotypes across the different populations in each North African country. In developing countries, over 70% of HDV patients progress to hepatic cirrhosis within two years [1, 2]. Such data have not been reported in North African populations. Hence, studies are needed to determine the subsequent risk of hepatic decompensation and liver-related death and to compare that with HBV mono infection among North Africans infected with HDV.

Risk factors for HDV infection in North Africa have not been studied and we did not identify any study showing an association between hepatitis D virus seroprevalence and HIV, HCV coinfection, intravenous drug use, and other HDV risk factors. However, North Africa has been considered a leading hub of drug trafficking and an area of high prevalence of HCV and HIV. A large cohort study carried out in Libya in 2014 showed a major increase in the acquisition of HIV, HBV (plus or minus HDV), and HCV, particularly among intravenous drug users [73]. This is in agreement with a recent hepatitis delta outbreak reported in ongoing epidemics of injection drug use in Eastern Europe and Russia [74]. Interestingly, new incident cases of HDV in Taiwan are no longer among intravenous drug users but among HIV-positive homosexual men, often presenting with liver flare-ups and syphilis independently of the use of successful antiretroviral therapy [75].

Several limitations are associated with our data analysis and should be considered. First, the collected studies were few, of moderate quality, and composed of small specific populations. The analyses are limited by the variability in quantity and quality of studies across countries. These studies may not be fully representative of the situation in the general populations of North Africa. Moreover, there is a geographical imbalance in the number of studies published. Very limited data were collected from certain countries, particularly Morocco and Algeria; thus, the overall estimated prevalence determined in our review may not be fully representative of the national and regional prevalence of HDV. Second, HDV infection is not clearly characterized in the searched studies and this could negatively reflect on the prevalence estimates of HDV in the North African populations. Third, the quality of laboratory testing lacks specificity, sensitivity, and uniformity, particularly for HBV, and few studies have used additional markers for detection of HBV infection, such as detection of HBV core antibodies or DNA testing. This could undermine the prevalence estimates of viral hepatitis from the collected data.

Despite the limitations of the results presented here, this review highlights the main aspects of HDV in North Africa and categorizes the prevalence of HDV in different populations. This review could be used to help identify which studies are needed for specific populations, at both the national and regional levels of North African countries. These results are relevant and topical for countries that are planning their national strategies against viral hepatitis. Better estimates are important for resources planning and will allow more accurate monitoring of the impact of future preventive and curative interventions.

Further studies are needed for a better understanding of the epidemiology of HDV infection in North Africa. The implications of immigration and population displacement (which have been accelerated by the recent political uprisings) on delta hepatitis are not known. A recent comprehensive study of African immigrants residing in Libya has shown high prevalence rates of HCV, HBV and HIV, which may influence the geo-epidemiology of viral hepatitis in North Africa and European countries, particularly those on the Mediterranean basin [76–79].

This systemic review highlights the endemicity of HDV in North Africa, which make it the second region in world following sub-Saharan Africa, with an estimated four million people infected with the virus. This makes it an important disease propagated by under-diagnosis and lack of management. Improving screening and diagnosis and broadening access to treatment should be apriority in tackling HDV in North Africa.

## Figures and Tables

**Table 1:** Prevalence of antihepatitis D virus and hepatitis D virus RNA in HBsAg-positive populations in North Africa.

**Year**	**Category**	**Tested (n)**	**Anti-HDV**		**HDV RNA**	
			n (%)	95% CI	n (%)	95% CI
**Egypt**						
2000	Out-patients	45	4 (9)	2.91-14.89	1(25)	19-34
2010	Liver disease patients	14	2 (14)	8.94-19.66	-	-
2010	Liver disease patients	30	4 (13)	8.52-18.08	1(25)	17-32
2013	Blood donors	170	8 (5)	1.58-7.82	-	-
2016	Hospital patients	121	10 (8)	2.16-14.44	3(30)	7-74

**Libya**						
2015	General population	346	2(2)	1.0-2.0	-	-
2012	Hospital patients	162	4 (3)	1.0-4.11	1(25)	5-68

**Tunisia**						
2013	General population	464	21(4.5)	1.56-7.44		
*2013 *	Liver disease patients	22	6(27.2)	9.87-44.53	2(33)	31-79
2013	Liver disease patients	55	10(18.1)	7.91-28.29	3(30)	29-71
2015	General population	1615	216(2)	1.21- 2.79	-	-
**Sudan**						
2015	Hospital patients	90	4(4.4)	1.75-7.05	-	-
2015	Hemodialysis patients	90	6(7.36)	3.86-10.86	-	-
2015	Pregnant women	90	3(3.3)	1.41-5.19	1(30)	32-69

**Mauretania**						
2008	Blood donors	447	78(19)	15-24	54(69)	52-78
2009	Pregnant women	109	14(13)	8-27	11(79)	42-91
2009	Hospital patients	162	29(18)	14-28	21(72)	51-86

**Algeria**						
2013	General population	296	12(4.0)	2.0-6.0	-	-

**Morocco**						
2010	General population	561	19 (3.0)	1.0-6.0	-	-

## Additional Points

*Research in Context*

*Evidence before This Study*. According to a literature search using PubMed, Embase, Scopus, Science Direct, Web of Science, and Google Scholar with the search terms “hepatitis delta virus,” “North Africa”, and the names of the countries there is no systematic review or meta-analysis on the prevalence of HDV in this region which has been published in the 20 years between January 1, 1998, and December 31, 2017. Most of the data published in this respect were carried out in Egypt, Tunisia, Mauritania, and Sudan. We identified no papers in which demographic or risk factors for HDV infection or a possible association between HDV infection and HIV status were analyzed.

*Added Value of This Study*. In this review, we systematically compiled all the available data on the epidemiology of HDV in North Africa. Our analysis indicates a large variation in the prevalence of HDV among the countries in this region. The highest prevalence rate was reported in Sudan, Mauritania, and Egypt, followed by Tunisia, Morocco, Algeria, and Libya. The published data do not enable us to draw a firm conclusion about the contribution of risk factors to the prevalence of HDV, and there is lack of specificity of the diagnosis and confirmation of HDV in the published data.

*Implications of All the Available Evidence*. HDV infection can have a large impact on the burden of liver diseases in North Africa, particularly in Sudan, Mauritania, and Egypt. Further studies are needed to better understand HDV in North Africa, and one requirement is standardization of specific tests for diagnosis and confirmation of HDV infection. Studies on HDV genotyping and risk factors (including intravenous drug use and immigration) are needed to formulate preventive and management strategies for HDV in Northern Africa.

## Supplementary Materials

Supplementary Materials Map of North African countries included in the study illustrating geographic locations and demographic features.
