# Supplementary material for: The Epidemiology of Hepatitis D Virus in North Africa: A Systematic Review and Meta-Analysis
Source: ScientificWorldJournal. 2018 Sep 26;2018:9312650. doi: 10.1155/2018/9312650 (PMC6178169; doi:10.1155/2018/9312650)
Supplement: Supplementary Materials — Map of North African countries included in the study illustrating geographic locations and demographic features. [file 9312650.f1.pptx]

## Slide 1
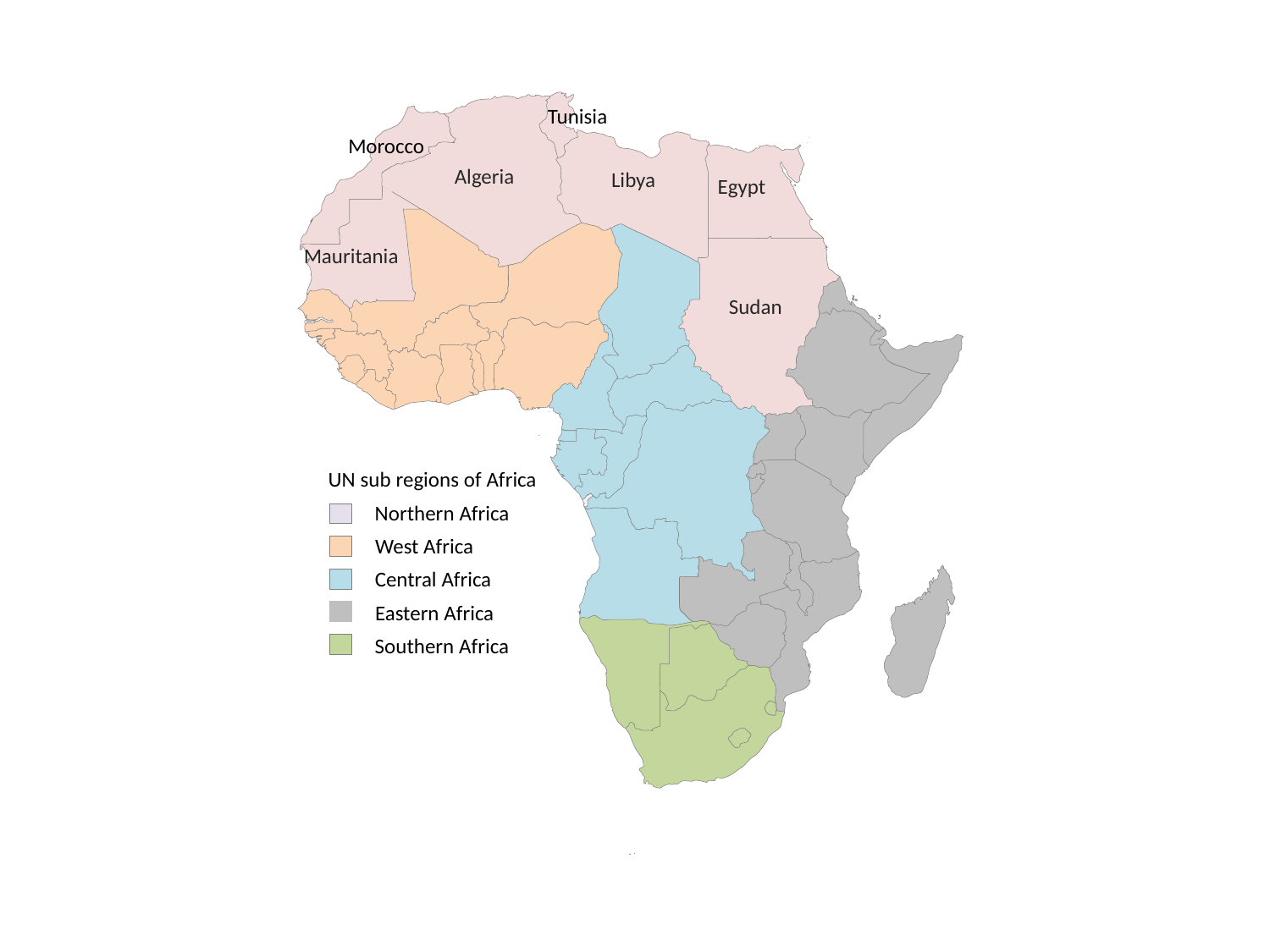

Algeria
Libya
Egypt
Mauritania
 Sudan
Tunisia
Morocco
UN sub regions of Africa
Northern Africa
West Africa
Central Africa
Eastern Africa
Southern Africa

## Slide 2
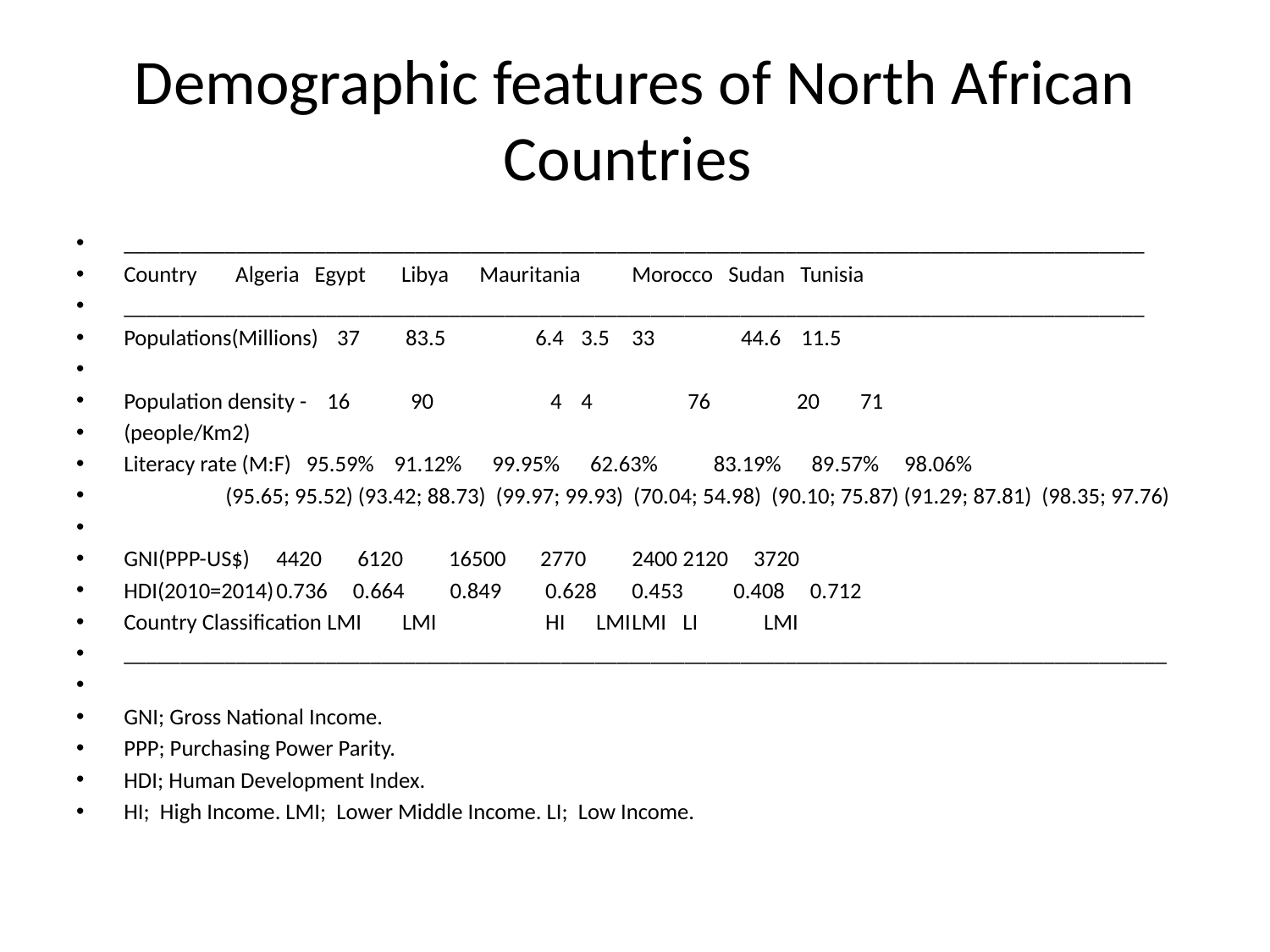

# Demographic features of North African Countries
___________________________________________________________________________________________
Country		 Algeria Egypt Libya	Mauritania 	Morocco Sudan Tunisia
___________________________________________________________________________________________
Populations(Millions)	 37 83.5	 6.4	3.5	33 44.6 11.5
Population density -	16 90	 4	4 	 76 20 71
(people/Km2)
Literacy rate (M:F) 95.59% 91.12% 99.95% 62.63% 83.19% 89.57% 98.06%
 (95.65; 95.52) (93.42; 88.73) (99.97; 99.93) (70.04; 54.98) (90.10; 75.87) (91.29; 87.81) (98.35; 97.76)
GNI(PPP-US$)	4420 6120 16500	 2770	2400	2120 3720
HDI(2010=2014)	0.736 0.664 0.849	 0.628	0.453	0.408 0.712
Country Classification	LMI LMI	 HI	 LMI	LMI	LI LMI
_____________________________________________________________________________________________
GNI; Gross National Income.
PPP; Purchasing Power Parity.
HDI; Human Development Index.
HI; High Income. LMI; Lower Middle Income. LI; Low Income.

## Slide 3
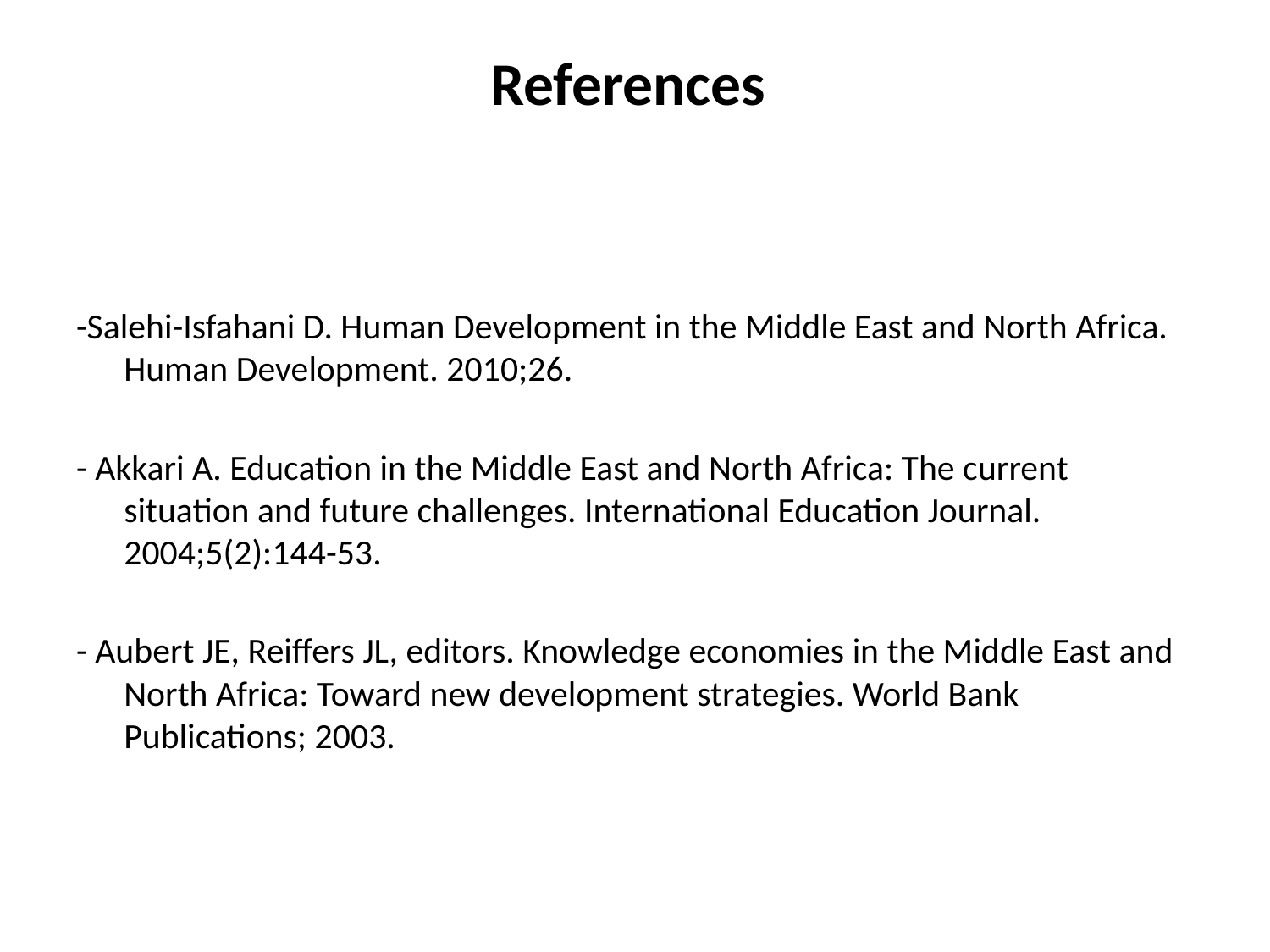

# References
-Salehi-Isfahani D. Human Development in the Middle East and North Africa. Human Development. 2010;26.
- Akkari A. Education in the Middle East and North Africa: The current situation and future challenges. International Education Journal. 2004;5(2):144-53.
- Aubert JE, Reiffers JL, editors. Knowledge economies in the Middle East and North Africa: Toward new development strategies. World Bank Publications; 2003.
